# Prevalence of naturally occurring NS5A resistance-associated substitutions in patients infected with hepatitis C virus subtype 1a, 1b, and 3a, co-infected or not with HIV in Brazil

**DOI:** 10.1186/s12879-017-2817-7

**Published:** 2017-11-13

**Authors:** Fernanda Malta, Karine Vieira Gaspareto, Gaspar Lisboa-Neto, Flair José Carrilho, Maria Cássia Mendes-Correa, João Renato Rebello Pinho

**Affiliations:** 1 Institute of Tropical Medicine, LIM-07, University of São Paulo, Av. Dr. Enéas Carvalho Aguiar, 500 – 2nd floor IMT-II, São Paulo, SP 05403-000 Brazil; 2 Department of Gastroenterology, University of São Paulo School of Medicine, São Paulo, Brazil; 3 Department of Infectious Diseases, University of São Paulo School of Medicine, São Paulo, Brazil; 4 Institute of Tropical Medicine, LIM-52, University of São Paulo, São Paulo, SP Brazil; 5 Hospital Israelita Albert Einstein, São Paulo, Brazil

**Keywords:** HIV/HCV coinfection, NS5A, DAA therapy, Resistance-associated substitutions

## Abstract

**Background:**

Non-structural 5A protein (NS5A) resistance-associated substitutions (RASs) have been identified in patients infected with hepatitis C virus (HCV), even prior to exposure to direct-acting antiviral agents (DAAs). Selection for these variants occurs rapidly during treatment and, in some cases, leads to antiviral treatment failure. DAAs are currently the standard of care for hepatitis C treatment in many parts of the world. Nevertheless, in Brazil, the prevalence of pre-existing NS5A RASs is largely unknown. In this study, we evaluated the frequency of naturally occurring NS5A RASs in Brazilian patients infected with HCV as either a monoinfection or coinfection with human immunodeficiency virus (HIV).

**Methods:**

Direct Sanger sequencing of the NS5A region was performed in 257 DAA-naïve patients chronically infected with HCV (156 monoinfected with HCV and 101 coinfected with HIV/HCV).

**Results:**

The frequencies of specific RASs in monoinfected patients were 14.6% for HCV GT-1a (M28 V and Q30H/R), 6.0% for GT-1b (L31F/V and Y93H), and 22.6% for GT-3a (A30K and Y93H). For HIV/HCV-coinfected patients, the frequencies of RAS were 3.9% for GT-1a (M28 T and Q30H/R), and 11.1% for GT-1b (Y93H); no RASs were found in GT-3a sequences.

**Conclusions:**

Substitutions that may confer resistance to NS5A inhibitors exist at baseline in Brazilian DAA-naïve patients infected with HCV GT-1a, −1b, and -3a. Standardization of RAS definitions is needed to improve resistance analyses and to facilitate comparisons of substitutions reported across studies worldwide. Therapeutic strategies should be optimized to efficiently prevent DAA treatment failure due to selection for RASs, especially in difficult-to-cure patients.

## Background

Due to their shared routes of transmission, co-infection with hepatitis C virus (HCV) and human immunodeficiency virus (HIV) is common worldwide, it is estimated that 2.3 million HIV-infected people also produce antibodies against HCV [1]. This population has long been considered a special at-risk population in terms of disease progression with subsequent mortality and a lower response to HCV therapies using interferon and ribavirin [2].

Currently, HCV-infected patients are treated with direct-acting antiviral agents (DAAs) using effective and well-tolerated completely oral regimens. DAAs target HCV non-structural proteins essential for genome replication and virion assembly, i.e. NS3/4A, NS5B, and NS5A. In addition, NS5A has also been implicated in host immune evasion [3]. To improve patient response to therapeutics and avoid viral resistance, new regimens consist of combinations of inhibitors of non-structural proteins NS3/4A, NS5B, and/or NS5A [4, 5].

In the era of these DAA therapies, the treatment response rates have not differed between HIV/HCV-coinfected and HCV-monoinfected patients [6]. HIV coinfection is no longer considered a risk factor for poor response to HCV treatment. Nevertheless, the presence of resistance-associated substitutions (RASs) prior to treatment may contribute to treatment failure, regardless of the presence of HIV infection [7]. A sustained virological response is a multifactorial event reliant on the therapeutic regimen and viral and host factors. Predictors of treatment failure include infection with HCV GT-1a and 3a, the presence of cirrhosis, and prior treatment with interferon-containing regimens [8].

NS5A substitutions have been identified in DAA treatment-naïve patients and may reduce the antiviral activity of NS5A inhibitors currently used in the clinic, such as daclatasvir, elbasvir, ledipasvir, ombitasvir and velpatasvir [9–12]. In Brazil, the interferon-free regimen for HCV treatment consists of a combination of sofosbuvir and daclatasvir, or sofosbuvir and simeprevir, with or without ribavirin [13].

Considering that there is little data on the prevalence of naturally occurring NS5A RASs in Brazil, the aim of the present study was to determine the frequency of natural NS5A RASs patients infected with HCV or HCV/HIV.

## Methods

### Study population

In this transversal study, a total of 257 DAA-naïve patients with chronic HCV-infections were enrolled. Of these patients, 156 were monoinfected (GT-1a: *n* = 41, GT-1b: *n* = 84, and GT-3a: *n* = 31) and 101 were coinfected with HIV/HCV (GT-1a: *n* = 77, GT-1b: *n* = 9, and GT-3a: *n* = 15). Patients were followed up at the Clinics Hospital of the School of Medicine of University of São Paulo.

HCV viral load of monoinfected patients was 6.17 Log UI/mL (IQR 5.78–6.51) and 6.31 Log UI/mL (IQR 6.02–6.71) for coinfected patients. At the time of this study, none of the enrolled patients had been treated with DAAs. However, 50.6% (79/156) of HCV monoinfected and 41.6% (42/101) of coinfected patients had previously been treated with PEGylated interferon-alpha (PEG-IFNa) and ribavirin (RBV), and, thus, were treatment-experienced without virological response. In addition, the majority of HCV/HIV co-infected patients was on antiretroviral therapy and thus has a good immune profile (data not shown).

### RNA extraction and NS5A amplification

Viral RNA was extracted from 200 μL of each plasma sample using QIAamp MinElute Virus Spin (Qiagen, Hilden, Germany) according to the manufacturer’s recommendations. cDNA synthesis and the first round of polymerase chain reaction (PCR) were performed using the SuperScript III One-Step RT-PCR system with Platinum Taq High Fidelity DNA Polymerase (Invitrogen™, Thermo Fisher Brand, Carlsbad, CA, USA). Next, a nested-PCR was performed using Platinum Taq DNA Polymerase (Invitrogen™, Thermo Fisher Brand, Carlsbad, CA, USA). NS5A genotype-specific primers and the amplification conditions for HCV GT-1a and GT-1b are described in Paolucci et al. [14] and, for HCV GT-3a, in previous work [15].

### Sequencing by Sanger method

Nested PCR products were purified using 4 μl of ExoSAP-IT (Affymetrix, Santa Clara, CA, USA) according to the manufacturer’s instructions. A cycle-sequencing reaction was performed using the BigDye® Terminator v3.1 Kit (Applied Biosystems, Thermo Fisher Brand, Carlsbad, CA, USA) with the same primers used in the nested PCR. Direct sequencing by the Sanger method was performed using the automatic sequencer ABI 3500 genetic analyzer (Applied Biosystems, Thermo Fisher Brand, Carlsbad, CA, USA).

### Sequence analysis

Electropherogram quality analyses were conducted and consensus sequences were obtained using the online software (http://asparagin.cenargen.embrapa.br/phph/). Nucleotide sequences were aligned against the reference genomes NC_004102 for GT-1a, AJ238799 for GT-1b, and D28917 for GT-3a using ClustalW integrated into Bioedit software (v. 7.0.8) [16]. In addition, all sequences were analyzed by geno2pheno[hcv] [17] to confirm amino acid substitutions and drug sensitivity. Substitutions at amino acid positions 28, 30, 31, and 93 in NS5A were considered RASs and have been previously reported as clinically relevant [18–23].

### Statistical analysis

The Fisher exact test was applied to assess an association between identified RASs and each patient group (HCV mono and HCV / HIV co-infected patients) according to HCV subtype, *p* values were calculated and considered statistically significant if *P* < 0.05. Analyses were conducted using SPSS software, version 15.0 (SPSS Inc., Chicago. IL, USA).

## Results

### Sequencing of the HCV NS5A region

The NS5A region was successfully sequenced from all 257 samples tested in this study and RASs were found in both treatment-naïve patient groups: HCV monoinfected and HIV/HCV coinfected.

Using Sanger-based sequencing, RASs were observed in 10% (22/257) of the amplified NS5A region sequences. Specifically, RASs were detected in sequences from 7.6% (9/118) of GT-1a-, 6.5% (6/93) of GT-1b-, and 15.2% (7/46) of GT-3a-infected patients. Population sequencing generated the sequence of the most common virus present in a sample (sensitivity ~20%).

Table 1 summarizes all detected amino acid substitutions (RASs and non-RASs) observed at NS5A amino acid residues 28, 30, 31, and 93. HCV GT-1a-monoinfected patients more frequently had NS5A RASs than those who were coinfected with HIV/HCV GT-1a (14.6% and 3.9%, respectively; Table 2), highlighting the increased prevalence of Q30H in HCV GT-1a-monoinfected patients.

**Table 1:** Frequencies of amino acid substitutions detected in HCV NS5A protein from mono-infected and HIV/HCV co-infected patients

Amino acid residues	HCV (*n* = 156)			HIV/HCV (*n* = 101)		
	1a (*n* = 41)	1b (*n* = 84)	3a (*n* = 31)	1a (*n* = 77)	1b (*n* = 9)	3a (*n* = 15)
M/L28^a^	**V (1; 2.4%)**	V (1; 1.2%)	–	–	–	
	I (1; 2.4%)	–	–	I (3; 3.9%)	–	
	L (1; 2.4%)	–	–	–	–	
	–	P (1; 1.2%)	–	–	–	
	–	–	–	**T (1; 1.3%)**	–	
Q/R/A30^a^	**H (4; 9.8%)**	–	–	**H (1; 1.3%)**	–	
	**R (1; 2.4%)**	–	–	**R (1; 1.3%)**	–	
	–	K (1; 1.2%)	**K (5; 16.1%)**	–	–	
	–	Q (3; 3.6%)	–	–	Q (1; 11.1%)	
	–	–	S (1; 3.2%)	–	–	S (1; 6.7%)
	–	–	–	L (1; 1.3%)	–	
L31	–	**F (1; 1.2%)**	–	–	–	
	–	**V (2; 2.4%)**	–	–	–	
Y93	–	**H (2; 2.4%)**	**H (2; 6.5%)**	–	**H (1; 11.1%)**	
Total (RAS)	14.6% (6/41)	6.0% (5/84)	22.6% (7/31)	3.9% (3/77)	11.1% (1/9)	0% (0/15)

*HCV* hepatitis C virus and *HIV* human immunodeficiency virus

^a^NS5A: M28 and Q30 are the dominant amino acids in GT-1a; L28 and R30 are the dominant amino acids in GT-1b; M28 and A30 are the dominant amino acids in GT-3a

Bold type represents the clinically relevant resistance-associated substitution (RAS). Data interpreted according to cited references [5, 23, 27, 33, 39–42]

**Table 2:** Frequency of HCV NS5A clinically relevant resistance-associated substitution (RAS) in mono-infected and HIV/HCV co-infected patients according to HCV subtype

	HCV 1a (*n* = 41)	HIV/HCV 1a (*n* = 77)	*p*-value^#^	HCV 1b (*n* = 84)	HIV/HCV 1b (*n* = 9)	*p*-value^#^	HCV 3a (*n* = 31)	HIV/HCV 3a (*n* = 15)	*p*-value^#^
RAS (+) (n, %)	6 (14.6%)	3 (3.9%)	0.063	5 (6.0%)	1 (11.1%)	0.467	7 (22.6%)	0 (0%)	0.078
RAS (−) (n, %)	35 (85.4%)	74 (96.1%)		79 (94.0%)	8 (88.9%)		24 (77.4%)	15 (100%)	

*HCV* Hepatitis C virus and *HIV* Human immunodeficiency virus

Clinically relevant resistance-associated substitution (RAS) interpreted according to cited references [5, 23, 27, 33, 39–42]

^#^Fisher’s exact test

### RASs in HCV-monoinfected patients

Among the HCV-monoinfected patients, RASs were observed in 11.5% (18/156). Among the HCV subtypes, 14.6% of RASs in GT-1a were M28 V and Q30H/R, 6.0% in GT-1b were L31F/V and Y93H, and 22.6% in GT-3a were A30K and Y93H. None of the NS5A sequences from HCV GT-1a harbored the Y93H variant (Table 1). Furthermore, in HCV subtype 1b sequences, P58S (2/84), E62H/E/P (3/84 were E62H, 2/84 were E62E, and 1/84 were E62P), and A92V/T (1/84 were A92V and 4/84 were A92T) polymorphisms were also detected.

Multiple amino acid substitutions were observed at a low frequency (<10%) in NS5A sequences. From monoinfected patients, GT-1a sequences had combinations of M28I + Q30H (1/41; 2.4%) and M28 L + Q30R (1/41; 2.4%), GT-1b had R30K + L31F (1/84; 1.2%), and GT-3a had S62 L + Y93H (1/31; 3.2%) and P58S + Y93H (1/31; 3.2%).

### RASs in HCV/HIV-coinfected patients

The RAS frequency in HCV/HIV-coinfected patients was 4% (4/101). Grouping by HCV subtypes, RASs were detected in 3.9% of GT-1a sequences (M28 T and Q30H/R) and 11.1% of GT-1b sequences (Y93H) (Table 1). Other polymorphisms also observed in GT-1a sequences were H58S/R/P (H58S was found in 1/77, H58R in 7/77, and H58P in 13/77) and E62D (5/77). GT-1b sequences contained P58S (3/9) and E62D/V (E62D was found in 1/9 and E62V in 1/9). Meanwhile, GT-3a sequences contained S62 T/Q (S62 T was found in 6/15 and S62Q in1/15).

In HCV/HIV-coinfected patients, multiple amino acid substitutions were detected only in GT-1a sequences. The combinations observed were M28 T + Q30L + H58S (1/77; 1.3%), M28I + H58R (2/77; 2.6%), and Q30R + H58P (1/77; 1.3%).

## Discussion

In this present study, the NS5A region was analyzed in HCV GT-1a, −1b, and -3a isolated from DAA treatment-naïve patients that were either monoinfected with HCV or coinfected with HCV/HIV from a major public hospital in São Paulo city.

Currently available HCV treatments are highly efficacious in and well-tolerated by most patients. Nevertheless, HCV resistance to DAAs has an important role in the failure of interferon-free treatment regimens and is a major challenge faced by future treatment strategies [18, 19]. Previous work has described the rapid selection of NS5A RASs, particularly at residues 28, 30, 31, and 93 [5].

The presence of NS5A RASs at baseline is associated with the virologic failure of DAAs. However, RASs have different effects based on the DAA regimen, viral genotype/subtype, and population characteristics (e.g. treatment-experience and cirrhosis status) [8].

NS5A RASs appear to have an impact on patient response to treatment, especially in those infected with HCV-1a and HCV-3a. Testing for baseline RASs is recommended for determining treatment duration in HCV GT-1a-infected patients who are being considered for therapy with elbasvir/grazoprevir. This testing is also recommended for patients infected with HCV GT-1a and GT-3a with cirrhosis (American Association for the Study of Liver Diseases guidelines) and in all patients before retreatment following a failed therapeutic regimen with DAAs [24–26].

In this present study, NS5A HCV sequences from HCV-monoinfected and HCV/HIV-coinfected patients revealed RASs were more frequent in GT-1a (7.6%; 9/118) than GT-1b sequences (6.5%; 6/93). This observation is contrary to previous studies [14, 27–29], and is likely a result of study differences in classification of RASs (e.g. inclusion of substitutions not previously shown to be relevant in clinical trials), as well as sample size. In addition, it is plausible that the different geographic origins of the samples may be involved in the resistance profiles described. The proportion of detectable pre-existing RASs reported in previous studies is 13% of cases in North America and 14% in Europe [30].

In this study, the amino acid substitutions M28 V and Q30H/R were observed within the GT-1a sequences from monoinfected patients. Previously published work has shown that M28 V confers low-level resistance to ombitasvir [19], while Q30H/R confers high-level resistance to daclatasvir, ledipasvir, and ombitasvir (fold change >100) [18, 23].

In sequences from GT-1b-monoinfected patients, the RASs L31F/V and Y93H were observed. Y93H confers different levels of resistance to approved NS5A inhibitors, including elbasvir and velpatasvir [30–32]. Finally, in GT-3a sequences from monoinfections, the Y93H and A30K substitutions were present at low to moderate frequencies. Hernandez et al. [33] observed that A30K and Y93H variants in NS5A reduce viral sensitivity to daclatasvir and ledipasvir, respectively. The clinical interpretation of these findings remains challenging since the impact of NS5A RAS to any DAA is multifactorial (e.g. drug regimen specific, cirrhosis or treatment-experienced patients without cirrhosis).

In regard to HCV/HIV-coinfected patients enrolled in this study, M28 T and Q30H/R variants were found in GT-1a isolates and may confer variable levels of resistance depending on the approved NS5A inhibitors used. Moreover, GT-1b isolates were found with the Y93H substitution, which is associated with resistance to daclatasvir, ombitasvir, and elbasvir (24–93-fold) [34], as well as ledipasvir (>1000-fold) [18, 19]. No RASs were identified in GT-3a isolates. Importantly, the presence of HIV appears to have had no influence on RAS selection.

Additionally, in this present work, the amino acid substitution P58S was observed in HCV GT-1b sequences from HCV-monoinfected and HCV/HIV-coinfected patients. While this substitution alone has been found to confer no to minimal resistance to NS5A inhibitors, it can increase viral resistance to NS5A inhibitors when in combination with certain NS5A RASs (e.g. L31 + Y93H) [19, 24, 35]. Moreover, multiple combinations of variants, consisting of two or three substitutions, were observed, but the clinical importance of these is currently unknown.

Special attention should be given to NS5A RASs that only minimally impair viral fitness and persist for long periods after treatment failure [36–38]. Another important point to emphasize is that NS5A inhibitors are currently used in the majority of treatment regimens for hepatitis C in different parts of the world, including Brazil.

Therefore, information on the frequency of NS5A RAS is very important as it can guide therapeutic strategies and diagnostic decisions by assessing resistance in candidates when treating hepatitis C in Brazil. Standardization of RAS definitions is needed to improve resistance analyses and to facilitate comparisons of substitutions between studies worldwide. To our knowledge, this is the first study on the presence of NS5A RASs in GT-3a strains from Brazil.

## Conclusion

In conclusion, this study found NS5A RASs were a natural occurrence in both HCV-monoinfected and HIV/HCV-coinfected patients that were DAA naive. A limitation of this study was the small sample size and the lack of follow-up on the subsequent clinical courses with DAAs. It is important to perform this analysis on patients from other regions to more thoroughly characterize the pattern of resistance across both the country of Brazil and continent of South America.

## Abbreviations

DAA: Direct-acting antiviral
GT: Genotype
HCV: Hepatitis C virus
HIV: Human immunodeficiency virus
RAS: Resistance-associated substitution
SVR: Sustained viral response
